# Deaths from Chloroform

**Published:** 1871-02

**Authors:** 


### DEATHS FROM CHLOROFORM.

Another of these accidents, where "no blame is attached to any party concerned," is chronicled in the *Hartford Evening Post.* Indeed, except in the matter of using chloroform in place of the less dangerous ether, all due precaution seems to have been taken. Chloroform was given in this case to allow the reduction of a dislocated humerus. Before administering it, the surgeon carefully examined the heart and lungs and found them apparently free from disease. The testimony of physicians present is that he used more than ordinary caution.

"Dr. ——— himself stated, in answer to inquiries, that the reason why he exercised his unusual caution was because of Mr. ———'s habits as to the use of alcoholic stimulants, whereby his constitution was impaired. Dr. ——— also stated, in answer as to what he considered the cause of death, that he thought that the chloroform was the immediate or exciting cause, but that death would not instantly have occurred without a predisposing cause, such as some disease of the heart or other vital organs which could not have been detected. He also answered that deaths from chloroform were of more or less frequent occurrence, and that even this year, Dr. Simpson, *the discoverer of chloroform* [the italics are ours], had a patient die, to whom he himself was administering it, and that it had repeatedly happened in the hands of the most eminent surgeons."—*Med. and Sur. Journal.*

